# Phenotypic Alteration of Neutrophils in the Blood of HIV Seropositive Patients

**DOI:** 10.1371/journal.pone.0072034

**Published:** 2013-09-09

**Authors:** Tom Cloke, Markus Munder, Philip Bergin, Shanthi Herath, Manuel Modolell, Graham Taylor, Ingrid Müller, Pascale Kropf

**Affiliations:** 1 Department of Immunology, Faculty of Medicine, Imperial College London, London, United Kingdom; 2 Third Department of Medicine (Hematology, Oncology, and Pneumology), University Medical Center Mainz, Mainz, Germany; 3 International AIDS Vaccine Initiative Human Immunology Laboratory, Faculty of Medicine, Imperial College London, London, United Kingdom; 4 Department of Cellular Immunology, Max-Planck-Institute for Immunobiology and Epigenetics, Freiburg, Germany; 5 Section of Infectious Diseases, Imperial College London, London, United Kingdom; Imperial College London, United Kingdom

## Abstract

We have recently identified a novel population of activated low-density granulocytes (LDGs) in peripheral blood mononuclear cells of HIV seropositive patients. LDGs have a similar morphology to normal density granulocytes (NDGs), but are phenotypically different. Here we measured the expression levels of different phenotypic markers of granulocytes in the blood of HIV seropositive patients at different stages of HIV infection to determine whether the phenotype of NDGs and LDGs are affected by disease severity. Our results reveal that the phenotype of NDGs, but not that of LDGs, varies according to the severity of the disease.

## Introduction

Neutrophils play a central role in the elimination of pathogens by using several strategies such as the production of reactive oxygen species, the release of antimicrobial peptides and neutrophil extracellular traps (NETs) (summarised in [1]). In HIV infections, both the number and the functions of neutrophils are impaired (summarised in [2]). Functional abnormalities of neutrophils include impaired phagocytosis and production of toxic oxygen species [2]. Furthermore, it has been recently shown that whereas NETs formation can capture and eliminate HIV, HIV can counteract this by inducing the production of IL-10 by dendritic cells and therefore inhibiting NETs formation [3].

Human neutrophils constitutively express arginase [4], an enzyme that catalyses the conversion of L-arginine into ornithine and urea [5], [6]. Recently, the metabolism of L-arginine by arginase has emerged as a crucial mechanism for the regulation of immune responses: increased catabolism of L-arginine by arginase results in the depletion of L-arginine from the microenvironment; since L-arginine is essential for efficient T cell activation, this decrease in L-arginine results in impaired T cell responses [7], [8], [9], [10]. Increased arginase activity has been described in malaria [11], tuberculosis [12], leishmaniasis [13], [14], [15] and HIV [16], [17].

We have recently shown that PBMCs from HIV seropositive patients with low CD4^+^ T cell counts expressed significantly more arginase activity as compared to patients with high CD4^+^ T cell counts or uninfected controls [16]. Higher arginase expression in PBMCs from HIV seropositive patients was associated with decreased levels of CD3ζ expression, a marker of T cell dysregulation [16]. The phenotype of arginase-expressing cells was identified as low-density granulocytes (LDGs) as these cells co-purify with PBMCs following density gradient centrifugation. This difference in density distinguishes this population from the remaining granulocytes that co-purify with the erythocyte fraction following density gradient centrifugation and thus have been named normal-density granulocytes (NDGs). LDGs purified from HIV+ patients display a similar morphology as NDGs, but have major phenotypic differences suggesting that LDGs were activated neutrophils that had degranulated and released arginase [17]. In the present study, we aim to determine whether the phenotype of NDGs and LDGs differs in different stages of HIV infection.

## Materials and Methods

### Subjects and samples

Twenty-one HIV seropositive (HIV+) treatment-naïve individuals were recruited from St Mary's Hospital (Table 1). Plasma HIV-1 viral RNA was quantified by real-time PCR (Bayer Quantiplex assay (bDNA) PCR test; lower detection level of 50 copies/mL). The standard T lymphocyte markers CD3, CD4, CD8 were determined by flow cytometry. The study was approved by the National Research Ethics Service (05/Q0410/93) and all individuals gave written, informed consent before participation.

**Table 1:** Clinical data.

Patients	Age	Sex (M/F)	CD4 count	Viral load
**1**	46	M	840	3.42
**2**	38	M	780	4.96
**3**	33	M	680	4.35
**4**	41	M	620	3.25
**5**	27	M	580	2.39
**6**	60	M	540	3.16
**7**	42	M	540	4.54
**8**	34	M	540	4.77
**9**	23	M	520	3.99
**10**	30	F	440	4.24
**11**	46	M	390	5.27
**12**	48	M	330	4.79
**13**	51	M	300	5.57
**14**	40	M	170	4.91
**15**	34	M	160	4.6
**16**	49	M	160	4.4
**17**	46	M	140	5.28
**18**	38	M	128	5.69
**19**	27	F	25	5.57
**20**	44	M	20	5.7
**21**	38	F	4	4.43

Age, sex, CD4^+^ T cell counts and viral load were recorded for HIV+ patients (n = 21).

Twenty ml of peripheral blood was collected in EDTA tubes and PBMCs were isolated by density gradient centrifugation on Histopaque®-1077 (Sigma). Neutrophils were isolated from the erythrocyte fraction by dextran sulphate sedimentation [17]. All experiments were performed on fresh cell, immediately after processing.

### Flow cytometry

The following antibodies were used: CD14^FITC^, CD15^PE^ (BD Pharmingen), Arginase1^Alexa Fluor^®^647^ (Hycult Biotechnology), CD11b^PerCP-eFluro710^, CD16^eFluro450^, CD33^PE-Cy7^ (eBioscience), CD13^APC-Cy7^ (Biolegend), CD66b^FITC^ and CD63^FITC^ (Beckman Coulter). 1×10^6^ PBMCs were incubated with FcR blocking reagent (BD Pharmingen) and the antibodies against extracellular markers were added directly to cells. Cells were washed after 20 min, fixed, permeabilised and anti-arginase I^Alexa FluorR 647^ was added to the cells for 20 min as described in [18]. Analysis was performed on an FACS Canto II (BD Bioscience) and results were analyzed using FlowJo v8.7 (Tree Star, Ashland, OR).

### Statistical analyses

Data were evaluated for statistical differences using a two-tailed Mann-Whitney test and a Spearman's rank test when appropriate (GraphPad Prism 5); differences were considered statistically significant at *p*<0.05. Results are expressed as median± SEM.

## Results

**Figure 1:**
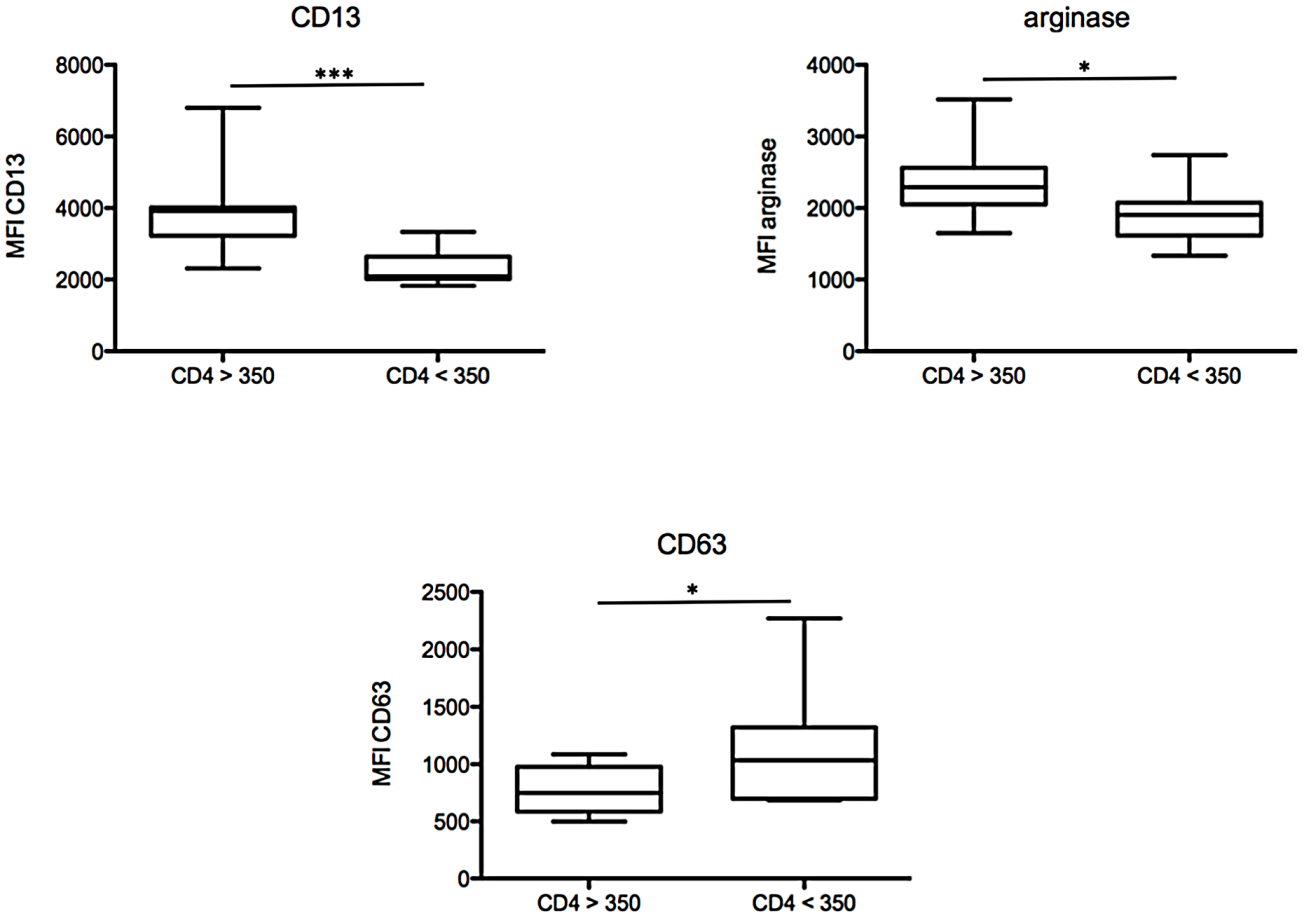
Phenotypic analysis of NDGs. NDGs were isolated from the blood of HIV+ patients with CD4^+^ T cell counts >350 (n = 11) or <350 cells/µL (n = 10) as described in materials and methods and the expression levels of phenotypic markers were determined by flow cytometry. Isotype controls: <1%. Statistical significance was determined by a two-tailed Mann-Whitney test. Box = interquartile range and median; whiskers = range.

We first analysed the phenotype of NDGs to determine whether it changes with increased disease severity, as measured by CD4^+^ T cell counts and viral load. We measured the expression levels of CD11b, CD13, CD15, CD16, CD33, CD63, CD66b and arginase on NDGs from treatment naïve HIV+ patients with high (≥350 cells/µL, CD4high) and low (<350 cells/µL, CD4low) CD4^+^ T cell counts. The division of patients based on a CD4^+^ T cell count <350 cells/µL was chosen because 1) once the CD4^+^ T cell count falls below 350 cells/µL differences in clinical outcome increasingly appear [19] and 2) the initiation of antiretroviral therapy is recommended once CD4^+^ T cell count falls to <350 cells/µL [20]. Our results show that the MFIs of CD13 and arginase are statistically significantly lower (*p* = 0.0008 and *p* = 0.0048, respectively) and that of CD63 significantly higher (*p* = 0.0346) in the blood of CD4low HIV+ patients (Figure 1, Table 2) as compared to CD4high HIV+ patients. No significant difference was observed in the expression levels of CD11b (*p* = 0.9159), CD15 (*p* = 0.3072), CD16 (*p* = 0.5495), CD33 (*p* = 0.3787) and CD66b (*p* = 0.6985) between CD4high and CD4low HIV+ patients (Table 2). Of note, the expression levels of CD11b, CD13, CD15, CD16, CD33, CD63, CD66b and arginase 1 were homogenous (Figure S1).

**Table 2:** Phenotype of NDGs.

	CD4high	CD4low	*p* value
	(median ± SEM)	(median ± SEM)	
**CD11b**	2684±767	3418±610	0.9159
**CD13**	3915±345	2091±152	**0.0008**
**CD15**	2270±693	3732±812	0.3072
**CD16**	10259±934	8731±743	0.5495
**CD33**	2114±196	1644±241	0.3787
**CD63**	751±57	1035±152	**0.0346**
**CD66b**	6326±771	6854±604	0.6985
**arginase**	2294±148	1904±124	**0.0448**

NDGs were isolated from the blood of HIV+ patients with CD4^+^ T cell counts >350 cells/µL (n = 11) or <350 cells/µL (n = 10) as described in materials and methods and expression levels of phenotypic markers were determined by flow cytometry.

**Figure 2:**
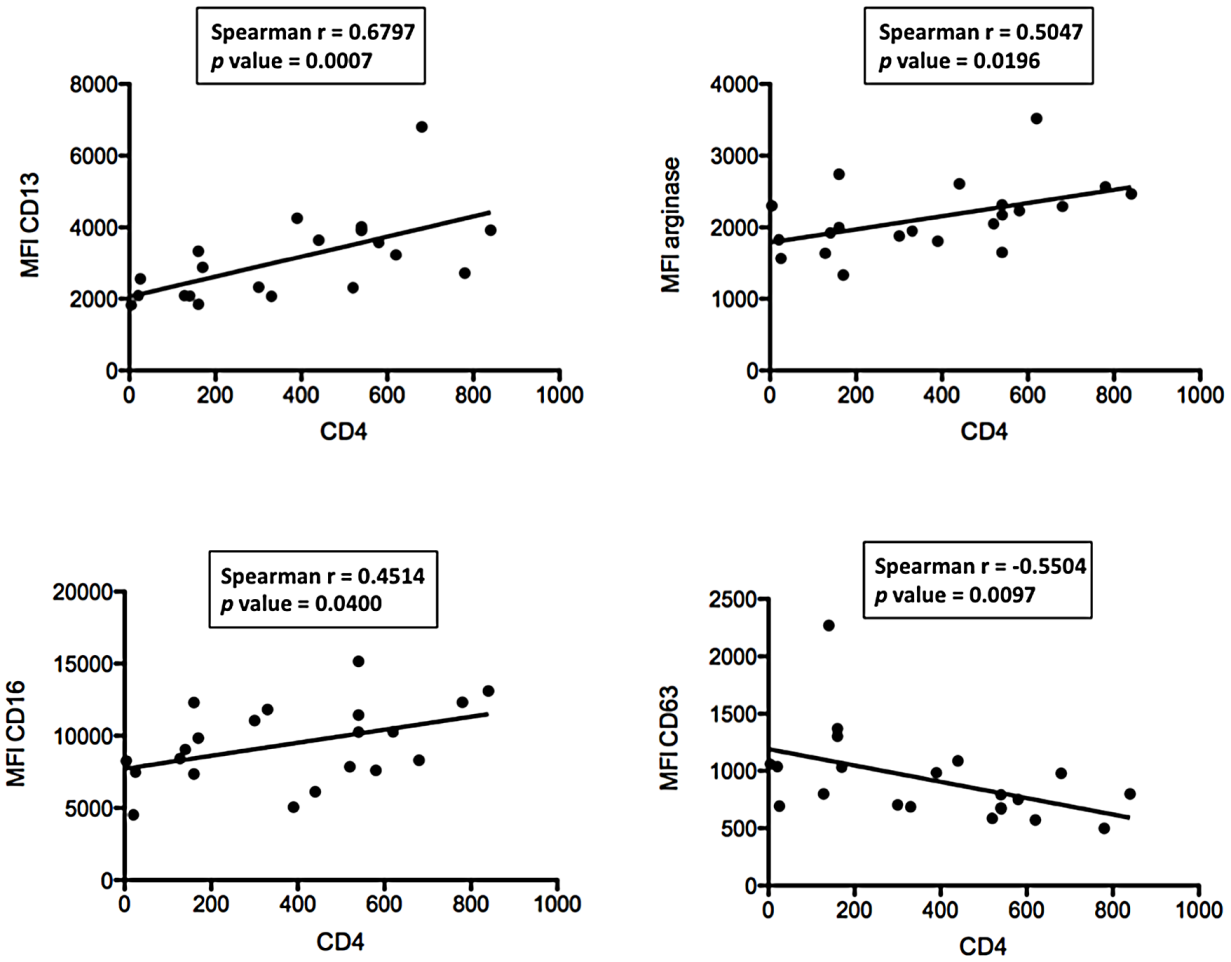
Correlation between CD4^+^ T cells and phenotypic markers. NDGs were isolated from the blood of HIV+ patients (n = 21) as described in materials and methods and the expression levels of phenotypic markers were determined by flow cytometry. Correlation between CD4^+^ T cell counts and phenotypic markers was determined by a Spearman's rank test.

To characterise further the association between these markers and disease severity, we plotted their MFI values against CD4^+^ T cell counts. As shown in Figure 2, there are statistically significant positive correlations between CD4^+^ T cell counts and the MFIs of CD13 (*p* = 0.0007), arginase (*p* = 0.0196) and CD16 (*p* = 0.0400); and a significant negative correlation between CD4^+^ T cell counts and the MFI of CD63 (*p* = 0.0097) (Figure 2, Table 3). No significant correlation was observed between CD4^+^ T cell counts and CD11b (*p* = 0.5122), CD15 (*p* = 0.0580), CD33 (*p* = 0.0825), and CD66b (*p* = 0.6064) (Table 3).

**Table 3:** NDGs: Correlation between CD4^+^ T cell counts and MFIs.

	Spearman r	*p* value
**CD11b**	−0.1522	0.5122
**CD13**	0.6797	**0.0007**
**CD15**	−0.3707	0.0980
**CD16**	0.4514	**0.0400**
**CD33**	0.3876	0.0825
**CD63**	−0.5504	**0.0097**
**CD66b**	−0.1213	0.6034
**arginase**	0.5047	**0.0196**

NDGs were isolated from the blood of HIV+ patients with CD4^+^ T cell counts >350 cells/µL (n = 11) or <350 cells/µL (n = 10) as described in materials and methods and the correlations between CD4^+^ T cell counts and phenotypic markers were determined by a Spearman's rank test.

**Figure 3:**
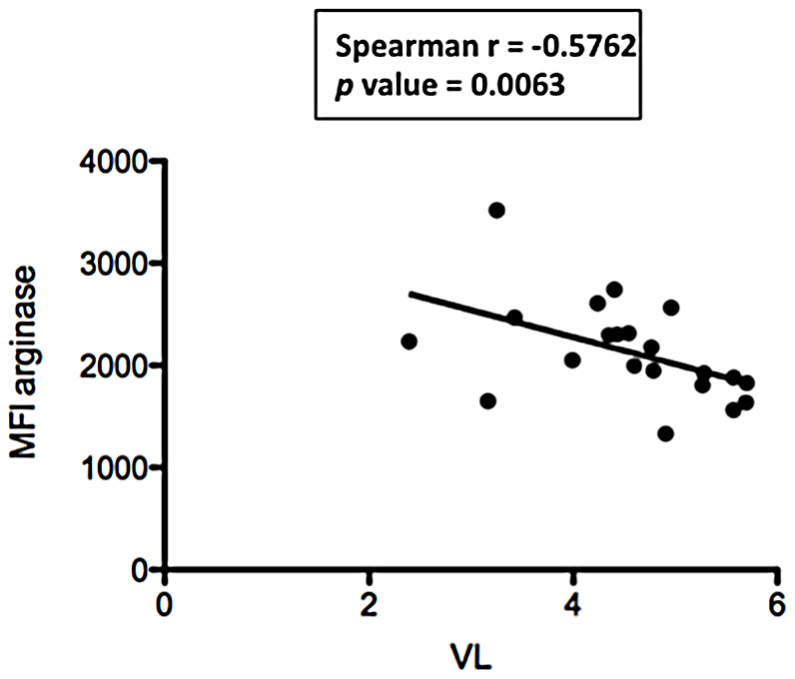
Correlation between viral load and phenotypic markers. NDGs were isolated from the blood of HIV+ patients (n = 21) as described in materials and methods and the expression levels of phenotypic markers were determined by flow cytometry. Correlation between viral load and phenotypic markers was determined by a Spearman's rank test.

Next, we plotted expression levels of CD11b, CD13, CD15, CD16, CD33, CD63, CD66b and arginase against another marker of disease severity, viral load. A statistically significant negative correlation between VL and arginase (*p* = 0.0063, Figure 3) and a trend towards significance between VL and CD13 (*p* = 0.0553, Table 4) were observed, all other correlations were not statistically significant (Figure 3, Table 4).

**Table 4:** NDGs: Correlation between viral loads and MFIs.

	Spearman r	*p* value
**CD11b**	0.1104	0.6337
**CD13**	−0.4242	0.0553
**CD15**	0.1241	0.5921
**CD16**	−0.2014	0.3814
**CD33**	−0.1845	0.4234
**CD63**	0.1358	0.5572
**CD66b**	0.1358	0.5574
**arginase**	−0.5762	**0.0063**

NDGs were isolated from the blood of HIV+ patients with CD4^+^ T cell counts >350 cells/µL (n = 11) or <350 cells/µL (n = 10) as described in materials and methods and thes correlation between viral load and phenotypic markers were determined by a Spearman's rank test.

These results suggest that in HIV+ patients, the phenotype of NDGs varies according to the severity of the disease.

Our previous results have identified a novel population of low-density granulocytes (LDGs) in the PBMCs of HIV+ patients that are morphologically similar, but phenotypically different from NDGs. Here, we determined whether the MFIs value differ in this population of LDGs and whether there was a correlation between markers of disease severity and the expression levels of CD11b, CD13, CD15, CD16, CD33, CD63, CD66b and arginase: MFIs values were similar between LDGs from CD4low and CD4high HIV+ patients (Table 5) and none of the correlations were statistically significant (Tables 6 and 7).

**Table 5:** Phenotype of LDGs.

	CD4high	CD4low	*p* value
	(median ± SEM)	(median ± SEM)	
**CD11b**	4413±649	5148±890	0.2907
**CD13**	2137±564	2368±366	0.9159
**CD15**	10012±1277	8262±769	0.3072
**CD16**	1528±1483	6932±1112	0.5035
**CD33**	3575±285	2768±304	0.1300
**CD63**	1474±178	1355±246	0.9717
**CD66b**	8899±2404	10198±1775	0.6472
**arginase**	1700±141	1533±123	0.2453

LDGs were isolated from the blood of HIV+ patients with CD4^+^ T cell counts >350 cells/µL (n = 11) or <350 cells/µL (n = 10) as described in materials and methods and the expression levels of phenotypic markers were determined by flow cytometry.

**Table 6:** LDGs: Correlation between CD4^+^ T cell counts and MFIs.

	Spearman r	*p* value
**CD11b**	−0.3507	0.1191
**CD13**	0.1028	0.6576
**CD15**	0.1502	0.5157
**CD16**	0.0072	0.9754
**CD33**	0.4039	0.0694
**CD63**	0.1158	0.6173
**CD66b**	−0.0134	0.9531
**arginase**	0.2738	0.7009

LDGs were isolated from the blood of HIV+ patients with CD4^+^ T cell counts >350 cells/µL (n = 11) or <350 cells/µL (n = 10) as described in materials and methods and the correlations between CD4^+^ T cell counts and phenotypic markers were determined by a Spearman's rank test.

**Table 7:** Correlation between viral loads and MFIs.

	Spearman r	*p* value
**CD11b**	0.2242	0.3286
**CD13**	0.0071	0.9755
**CD15**	−0.2007	0.3830
**CD16**	−0.0084	0.9710
**CD33**	−0.3248	0.1509
**CD63**	−0.0364	0.8756
**CD66b**	0.0948	0.6826
**arginase**	−0.4722	0.2235

LDGs were isolated from the blood of HIV+ patients with CD4^+^ T cell counts >350 cells/µL (n = 11) or <350 cells/µL (n = 10) as described in materials and methods and the correlations between viral load and phenotypic markers were determined by a Spearman's rank test.

These results suggest that the phenotype of NDGs, but not that of LDGs varies according to the severity of the disease.

### Phenotype of NDGs and LDGs in CD4low HIV+ patients

**Figure 4:**
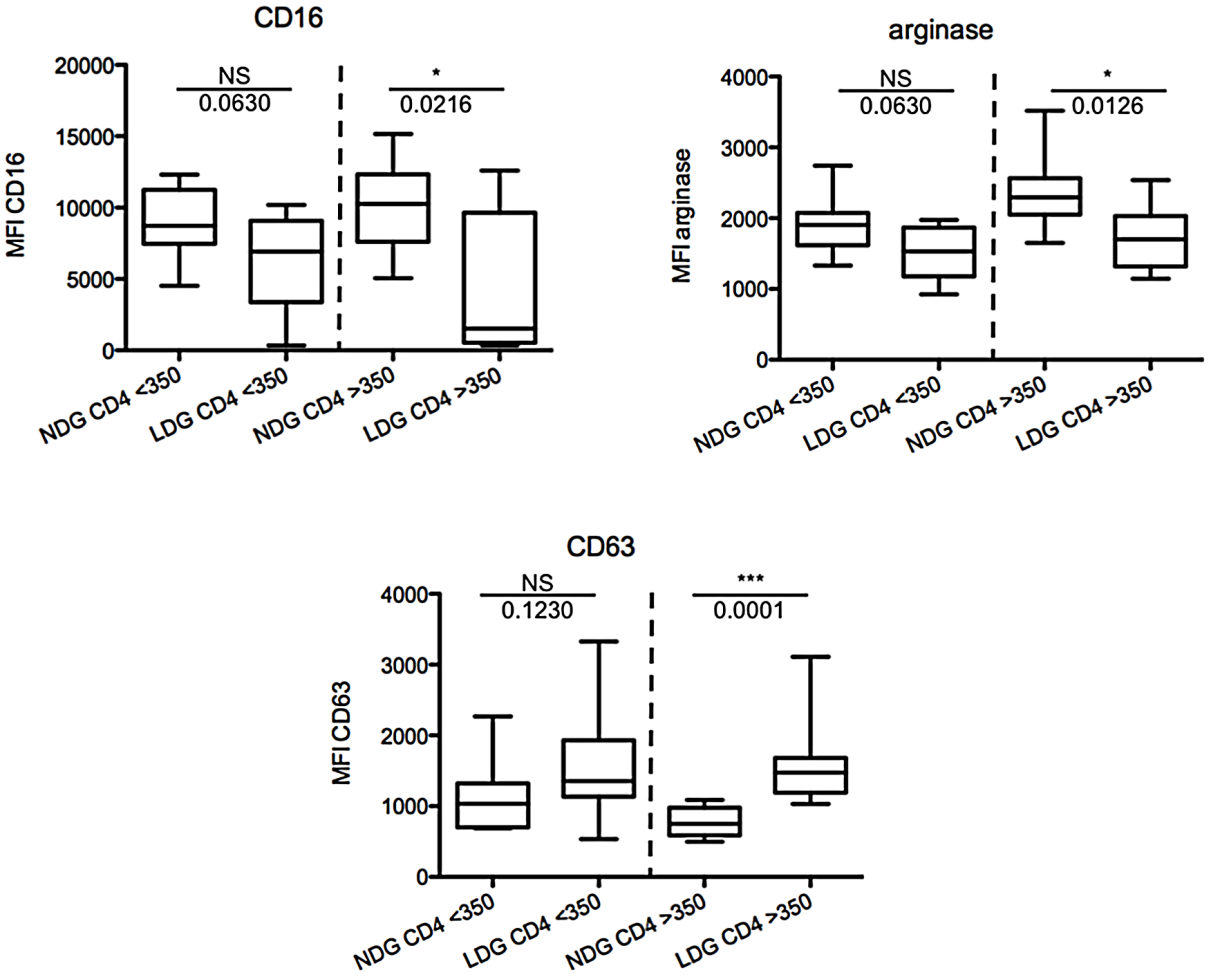
Phenotypes of NDGs and LDGs in CD4low and CD4high HIV+ patients. PBMCs and NDGs were isolated from the blood of HIV+ patients with CD4^+^ T cell counts >350 (n = 11) or <350 cells/µL (n = 10) as described in materials and methods and the expression levels of phenotypic markers were determined by flow cytometry. Isotype controls: <1%. Statistical significance was determined by a two-tailed Mann-Whitney test. Box = interquartile range and median; whiskers = range.

We have previously shown that LDGs are phenotypically different from NDGs, as they express increased levels of CD11b, CD15, CD33, CD66b, CD63 and decreased levels of CD16 and arginase 1 [17], suggesting that these cells are activated neutrophils that have degranulated and therefore change their density. Indeed, the results presented in Figure 2 suggest that NDGs get progressively more activated with increased disease severity, as measured by CD4^+^ T cell counts. In the next step, we assessed how the phenotype of NDGs differs from that of LDGs in CD4high and CD4low HIV+ patients. As shown in Figure 4 and Tables 8 and 9, the expression levels of CD16, arginase and CD63 are significantly different between LDGs and NDGs from CD4high HIV+ patients, but are similar in CD4low patients. Differences between CD11b, CD13 and CD66b MFIs remain non-significant and differences between CD15 and CD33 remain significant in CD4low and CD4high HIV+ groups.

**Table 8:** LDGs and NDGs in HIV+ patients in CD4low HIV+ patients.

	LDGs	NDGs	*p* value
	(median ± SEM)	(median ± SEM)	
**CD11b**	5148±890	3418±610	0.2176
**CD13**	2368±366	2091±152	0.9705
**CD15**	8262±769	3732±812	**0.0015**
**CD16**	6932±1112	8731±743	0.0630
**CD33**	2768±304	1644±241	**0.0288**
**CD63**	1355±246	1035±152	0.1230
**CD66b**	10198±1775	7671±604	**0.0355**
**arginase**	1533±123	1904±124	0.0630

LDGs and NDGs were isolated from the blood of HIV+ patients with CD4^+^ T cell counts <350 cells/µL (n = 10) as described in materials and methods. Expression levels of phenotypic markers were determined by flow cytometry.

**Table 9:** LDGs and NDGs in HIV+ patients in CD4high HIV+ patients.

	LDGs	NDGs	*p* value
	(median ± SEM)	(median ± SEM)	
**CD11b**	4413±694	2684±767	0.5994
**CD13**	2137±564	3915±345	0.1007
**CD15**	10012±1277	2270±693	**0.0003**
**CD16**	1528±1483	10259±934	**0.0216**
**CD33**	3575±285	2114±196	**0.0016**
**CD63**	1474±178	751±57	**0.0001**
**CD66b**	8899±1775	7697±604	0.1679
**arginase**	1700±141	2294±150	**0.0126**

Ldgs And Ndgs Were Isolated From The Blood Of Hiv+ Patients With Cd4+ T Cell Counts >350 Cells/µL (N = 11) As Described In Materials And Methods. Expression Levels Of Phenotypic Markers Were Determined By Flow Cytometry.

## Discussion

We have previously shown that arginase activity was significantly increased in the blood of CD4low HIV+ patients as compared to CD4high and healthy controls [16]. The phenotype of arginase-expressing cells in the PBMCs of HIV+ patients are a subset neutrophils, which were classified as low-density granulocytes (LDGs) [16], [17]. These cells have a similar morphology as normal density granulocytes (NDGs) [17]. However, LDGs differ from NDGs as i) they co-localise with the PBMCs and not the erythrocytic fractions, suggesting that their density is lower; ii) they express different levels of phenotypic markers of neutrophils. In addition, our results show that the frequency of LDGs is significantly higher in HIV+ patients with low CD4^+^ T cell counts and correlates with markers of disease severity in HIV+ patients [17]. We have already shown that the cells expressing arginase in PBMCs from HIV+ patients are LDGs, as they express CD15, but not CD14, and that the frequency of these cells increases with disease severity [16].

Our results show that there is no difference between the phenotype of LDGs from CD4low and CD4high HIV+ patients; however, there are clear phenotypic differences in the expression levels of CD13, CD63 and arginase in NDGs from HIV+ patients with low CD4^+^ T cell counts. Increased activation of neutrophils in HIV+ patients has already been described [21]. In this study, the expression levels of CD11b were shown to be increased on neutrophils from HIV+ patients as compared to HIV- controls. In our study we assessed whether the level of neutrophils' activation changes with disease severity, rather then comparing it to healthy controls. Our results are in agreement with the study by Elbim et al., as we did not find a change in CD11b expression on NDGs with lower CD4^+^ T cell counts.

Our results suggest that during the course of HIV infection, there is no progressive activation of LDGs, as we found no correlation between CD4^+^ T cell counts or viral load and the expression levels of CD11b, CD13, CD15, CD16, CD33, CD63, CD66b and arginase 1. However, our results suggest that NDGs become progressively and systemically more activated and more degranulated, since decreased expression levels of CD13, CD16 and arginase and increasing expression levels of CD63 on NDGs correlate with decreasing CD4^+^ T cell counts. The degree of neutrophil activation is regulated by the intensity of the activating signal and occurs sequentially: 1) secretory granules; 2) gelatinous granules; 3) specific and 4) azurophilic granules. Arginase is found in gelatinous granules [22] and azurophilic granules [4] and upregulation of CD63 on neutrophils coincides with the release of azurophilic granule [23], [24]. Therefore, our result showing that CD63 is expressed at increased levels and arginase at lower levels in NDGs from patients with low CD4^+^ T cells suggest that NDGs get progressively more activated with increased disease severity. We hypothesise that as a result of degranulation, NDGs will change their density and become LDGs, and will be collected in the PBMC fraction following density gradient purification. Our results suggest that NDGs are activated and have already, at least partially, released azurophilic granules, as shown by increased CD63 and decreased arginase expression levels in CD4low HIV+ patients. Furthermore, our results show that the MFIs of CD16, CD63 and arginase are significantly different between LDGs and NDGs in CD4high HIV+ patients, but not any more in CD4low HIV patients; suggesting that there are less differences between the phenotype of LDGS and NDGs in CD4low as compared to CD4high HIV+ patients.

The origins of LDGs as well as the signals resulting in the degranulation of neutrophils remain unclear in HIV+ patients and we have not been able to activate NDGs to become LDGs. Stimulation of neutrophils with Phorbol 12-myristate 13-acetate (PMA) and/or N-formyl-methionyl-leucyl-phenylalanine (fLMP) has been described previously ([23]: whereas these stimuli result in activation and degranulation of neutrophils, the phenotype of these activated neutrophils differs from that of LDGs we described [17], as CD13 and CD16 are both downregulated on activated NDGs.

Further work into novel markers of immune suppression, such as activation of granulocytic cells, is warranted as this may result in improvement in the clinical management of patients with HIV infection through: 1) better evaluation of disease severity (including the stage and rate of progression of the disease) and 2) informing the timing and choice of treatment initiation so as to minimise morbidity associated with opportunistic infections, drug resistance and medication side effects.

## Supporting Information

Figure S1 **Phenotypic analysis of NDGs.** NDGs were isolated from the blood of HIV+ patients with CD4^+^ T cell counts >350 (n = 11) or <350 cells/µL (n = 10) as described in materials and methods and the expression levels of phenotypic markers were determined by flow cytometry. Isotype controls: <1%. Statistical significance was determined by a two-tailed Mann-Whitney test. Box = interquartile range and median; whiskers = range.
(TIF)
