# Supplementary figures and images for: Phenotypic Alteration of Neutrophils in the Blood of HIV Seropositive Patients
Source: PLoS One. 2013 Sep 9;8(9):e72034. doi: 10.1371/journal.pone.0072034 (PMC3767740; doi:10.1371/journal.pone.0072034)

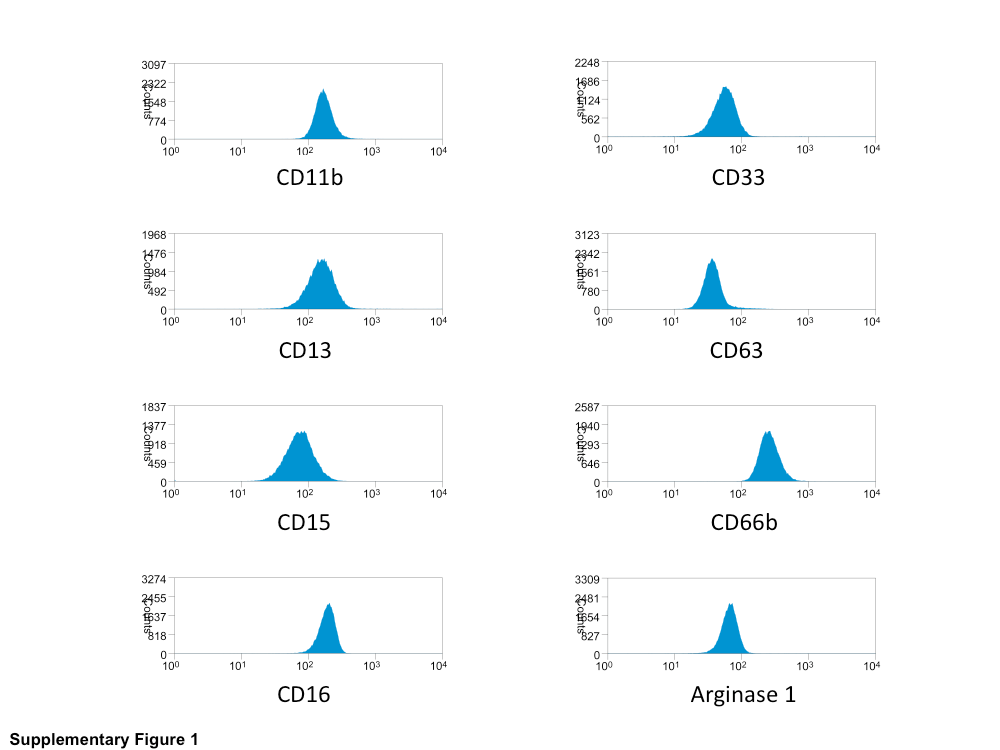

Supplement: Figure S1 — Phenotypic analysis of NDGs. NDGs were isolated from the blood of HIV+ patients with CD4+ T cell counts >350 (n = 11) or <350 cells/µL (n = 10) as described in materials and methods and the expression levels of phenotypic markers were determined by flow cytometry. Isotype controls: <1%. Statistical significance was determined by a two-tailed Mann-Whitney test. Box = interquartile range and median; whiskers = range. (TIF) [file pone.0072034.s001.tif]
